# The Best Time for Administering Medicines?

**Published:** 1885-10

**Authors:** 


### The Best Time for Administering Medicines ?

Before or after meals? Such is the question often asked of the doctor, but the answer is not always ready. The *Midland Medical Miscellany* answers it as follows: Medicines that are irritating should be given after meals, when the stomach is full, viz ; the salts of copper, zinc, iron and arsenic in large doses. Small doses, intended to act on the stomach terminals of the vagi, must be given when the organ is empty. Chemical reasons also have their influence, thus, oxide and nitrate of silver, intended for local action, should appear in the stomach during its period of inactivity, lest, at other times, chemicals reactions destroy the special attributes for which these remedies are prescribed. Iodine and the iodides further illustrate this point. Given on an empty stomach they promptly diffuse into the blood, but if digestion is going on, the acids and starch form products of inferior activity, and thus the purpose which they are intended to subserve is defeated. Substances prescribed to have alvine action on the mucous membrane, or for prompt diffusion unaltered, are preferably given before meals. The condition of the stomach veins after meals is such as to lessed the activity of diffusion of poisons, and hinders their passage through the liver. It follows that active medicaments in doses near the danger-line, are more safely administered after meals.

When shall acids and alkalies be given, before or after meals? First, as to acids. When acids are prescribed with the view to check the excessive formation of the acids of the gastric juice, they may be given before meals—as, by the laws of osmosis, they will determine the glandular flow of the alkaline constituents of the blood. The same reasoning would hold good the alkaline condition of the blood is in excess; osmosis being favored, the acid would reach the blood more readily. Second, as to alkalies. These may be given just before meals, when the acid-forming materials in the blood diffuse into the stomach glands, and after digestion is completed, when the alkalies diffuse directly into the bloyd, without interference from the contents of the stomach. An alkali taken during the time when the reaction of the stomach juices should be strongly acid, must necessarily hinder, if not arrest, the digestive process for the time being. The metalic salts—notably corrosive sublimate, alcohol, tannin, and some other agents—impair or destroy the ferment, or digestive power, pepsin. Wine that is intended to act as food, is most beneficial when taken slowly during the course of the meal. The objection as regards the ill efiect of alcohol on pepsin, is not applicable here except to the stronger spirituous wines in large quantities, for the ordinary medicinal wines do not have sufficient alcoholic strength to injure this ferment. Iron, phosphates, cod liver oil, malt and similar agents should, as a rule, go with food through the digestive process, and with the products of digestion enter the blood.—*American Medical Digest.*

